# Fermentation with Pectin Trans-Eliminase to Reduce Cadmium Levels in Nacional and CCN-51 Cocoa Bean Genotypes

**DOI:** 10.3390/plants14162553

**Published:** 2025-08-16

**Authors:** Wiston Javier Morales-Rodriguez, Jaime Morante-Carriel, Mercedes Carranza-Patiño, Darko Ormaza-Vásquez, María Concepción Ayuso-Yuste, María Josefa Bernalte-García

**Affiliations:** 1 Faculty of Industry and Production Sciences, Quevedo State Technical University, Av. Quito Km. 1 1/2 Vía a Santo Domingo de los Tsáchilas, Quevedo 120501, Ecuador; wmorales@uteq.edu.ec; 2 Escuela de Ingenierías Agrarias, Universidad de Extremadura, Avda. Adolfo Suárez s/n, 06007 Badajoz, Spain; bernalte@unex.es; 3 Plant Proteomics and Functional Genomics Group, Department of Biochemistry and Molecular Biology and Soil and Agricultural Chemistry, Faculty of Science, University of Alicante, Carretera San Vicente del Raspeig s/n, San Vicente del Raspeig, 03690 Alicante, Spain; jaime.morante@ua.es; 4 Faculty of Forestry and Agricultural Sciences, Quevedo State Technical University, Av. Quito Km. 1 1/2 Vía a Santo Domingo de los Tsáchilas, Quevedo 120501, Ecuador; mcarranza@uteq.edu.ec; 5 Faculty of Engineering Sciences, Quevedo State Technical University, Av. Quito Km. 1 1/2 Vía a Santo Domingo de los Tsáchilas, Quevedo 120501, Ecuador; darko.ormaza2017@uteq.edu.ec; 6 Instituto Universitario de Investigación de Recursos Agrarios (INURA), Universidad de Extremadura, Avda. de la Investigación s/n, Campus Universitario, 06006 Badajoz, Spain

**Keywords:** postharvest processing, chemical contamination, food safety, cocoa quality, cadmium

## Abstract

Cocoa represents a crucial source of income in coastal regions of Ecuador, where the product is exported for the production of high-value chocolates. However, elevated levels of cadmium (Cd) in cocoa beans, attributable to volcanic soils, have the potential to impede international trade, particularly in accordance with European Union regulations. The main objective of this study was to reduce Cd concentrations in cocoa beans of two genotypes, Nacional and CCN-51, by applying different doses of pectin trans-eliminase (PTE) enzyme during the fermentation process in conjunction with mucilage washing techniques, pre-drying resting periods, and various drying methods. To this end, a Taguchi orthogonal design (L9) was employed to evaluate nine treatments per genotype, complemented with two controls. The most efficacious treatment for Nacional was identified as T7, involving a 0.30 mL·kg^−1^ PTE dose, the absence of mucilage washing, a 48 h resting period, and drying in a marquee. This treatment resulted in a 68.6% reduction in Cd concentration (from 0.28 to 0.09 mg·kg^−1^). For CCN-51, T3 (0.10 mL·kg^−1^ PTE, complete washing, 48 h resting, and splint drying) yielded a 26.4% reduction in Cd (from 0.42 to 0.31 mg·kg^−1^). It is noteworthy that none of the treatments exceeded the EU regulatory threshold of 0.8 mg·kg^−1^. A physico-chemical analysis was conducted, which revealed significant treatment effects on pH (ranging from 5.63 to 6.85) and acidity (0.02% to 0.03%). Sensory evaluation indicated enhancements in cocoa and nutty flavors, along with a reduction in undesirable astringency and bitterness, particularly in Nacional samples. The findings of this study demonstrate that the combination of enzyme-assisted fermentation and optimized postharvest techniques represents a pragmatic approach to the mitigation of cadmium in cocoa, while simultaneously preserving or enhancing product quality.

## 1. Introduction

*Theobroma* is a genus in the *Sterculiaceae* family containing 22 species found in wet Neotropical woods [1]. *Theobroma cacao* L., which is used to make chocolate and other goods resulting from its processing, has been grown since the pre-Columbian period [2,3]. Cacao’s natural spread in Latin America expanded from the Amazon region to southern Mexico, and it is now also cultivated in some countries in Africa and Asia.

Cocoa is one of the most important agricultural products in the world due to the wide diversity of products in the chocolate industry and the resulting by-products, which have high nutritional value [4,5,6]. It is also considered a superfood due to its high antioxidant capacity and its vitamin and mineral content, as well as functional compounds such as polyphenols. In addition, it has secondary metabolism compounds, which are central nervous system stimulants and are of medical and pharmacological interest, such as caffeine, theobromine, and theophylline, and gentisic acid, which is antirheumatic and analgesic [7]. The global cocoa production was 4.9 million tons in 2022/2023, grown mainly in Côte d’Ivoire and Ghana, while Ecuador produced 0.32 million tons, representing 7% of the world total [8]. In 2023, Ecuador achieved remarkable success in cocoa production, exceeding 400,000 tons and generating revenues of more than USD 1 billion, representing 1.97% of the national GDP [9].

There is a higher demand for cocoa produced on certified organic cocoa farms in developed countries [10]. Latin America produces 70% of the world’s organic cocoa, with producers in Ecuador, the Dominican Republic, and Venezuela standing out. Ecuador is the main producer and exporter of fine aroma cocoa [11,12]. Many cocoa transnationals are currently in Ecuador [13], although small producers have managed to achieve the required standards and obtain organic cocoa certification. According to the data mentioned in the “Report of the Analysis of the Determinants of Cocoa Productivity” by the Ministry of Agriculture of Ecuador (MAGAP), the area planted in 2023 was 609,750 hectares, with a production of 362,727 tons, giving a yield of 0.73 tons·ha^−1^. Of this figure, 80% corresponds to the production of fine or flavor cocoa and 20% to the production of the CCN-51 cultivar; it is estimated that 90% of the renewed area is planted with CCN-51 [14].

In order to produce high-quality cocoa, the product must meet internationally accepted standards. The European Union (EU) and other countries have set limits on the amount of Cadmium (Cd) allowed in cocoa and cocoa products marketed in their regions. EU Regulation 488/2014 sets a maximum tolerable Cd limit of 0.8 mg·g^−1^ for chocolate with a cocoa solids content of 50% or more. Some buyers use this criterion to assess the Cd content of the cocoa beans they purchase in order to ensure the quality of the final products, as the EU has set maximum limits for Cd in chocolate products, but has not established specific restrictions for cocoa beans as a raw material [15]. The use of the enzyme pectin trans-eliminase (PTE) in the fermentation of cocoa beans promotes the breakdown of mucilage, due to its effectiveness in decomposing the components of the plant cell wall, producing butanol, propanol, and ethanol, acids such as acetic, lactic, succinic, and butyric acids, as well as carbon dioxide, ketones, aldehydes, and esters [16]. This enzyme facilitates the degradation or liquefaction of the cell wall; it induces the release of heavy metals, preventing Cd from penetrating the cocoa beans. Therefore, the use of this enzyme can help reduce the concentration of Cd in cocoa, preventing its accumulation in the human body, which causes serious health problems [17]. The presence of Cd in cocoa may be due to the high concentration of this heavy metal in some soils of volcanic origin in cocoa-producing areas of Ecuador [18,19,20].

In this context, it is hypothesized that the utilization of PTE during cocoa bean fermentation, in conjunction with optimized postharvest practices such as mucilage washing, pre-drying, resting time, and drying methods, can result in a substantial reduction in cadmium (Cd) levels without compromising the quality of the beans. In order to test this hypothesis, the study was conducted in Mocache canton, a key cocoa-producing region in the Ecuadorian province of Los Ríos, with the following objectives: (i) to evaluate the effects of different PTE enzyme concentrations; (ii) to assess the impact of postharvest variables on Cd reduction; and (iii) to determine their influence on physico-chemical and sensory characteristics. The objective of this approach is to furnish a pragmatic strategy for cadmium mitigation and quality enhancement in cocoa production, thereby benefiting local producers and aligning with international safety standards.

## 2. Results

### 2.1. Cutting Test

The results of the cutting test are shown in Table 1. A detailed description of the treatments applied, in terms of enzyme concentration, type of washing, and drying (T1–T9), as well as the characteristics of the controls (Control 1 and Control 2), is shown in the Experimental Design section. In the case of Nacional cocoa, significant differences were found in the variables Good fermentation, Medium fermentation, and Low fermentation. No significant differences were found for the remaining variables. The percentage of Good fermentation (GF) ranged from 78 to 88%, with Control 2 having the highest percentage of beans with good fermentation and T3 treatment having the lowest percentage (enzyme dose 0.10 mL·kg^−1^ of cocoa, complete washing, rest in pre-drying for 48 h, and drying on a splint). With regard to Medium fermentation (MF), the interval ranged between 5 and 15%; from 0 to 10% for Low fermentation (LF) and Over fermentation (OF) presented low rates, no higher than 3%, with T1, T2, and T6 being the treatments that did not show Over fermentation (with enzyme dose 0.10 or 0.20 mL·kg^−1^ of cocoa). The highest percentage of Slaty (S) beans was 3% in treatments T1 and T3 (enzyme dose 0.10 mL·kg^−1^ of cocoa), and 0% for T2 and T4 (enzyme dose 0.10 or 0.20 mL·kg^−1^ of cocoa, incomplete or without washing, rest in pre-drying for 24 h). Treatments T2, T3, T5, T8 (different enzyme doses and postharvest conditions), and Control 1 (mechanical mucilagination) showed the presence of mold (M) ranging from 1 to 2% of the beans, while treatments T3 and T8 showed insect damage (ID).

In CCN-51 cocoa, significant differences were found only in the variable Medium fermentation, with two levels of significance; the rest of the variables did not show significant differences. GF ranged from 77 to 90%, with treatments T7 (enzyme dose 0.30 mL·kg^−1^ of cocoa, without washing, rest in pre-drying for 48 h, and drying on a marquee) and Control 2 (producer local control) having the highest percentage of beans with this level of fermentation, while treatment T1 had the lowest percentage. For MF, the values ranged between 3 and 12%, and for Low fermentation, between 2 and 5%. With regard to OF, the maximum value was 1% for treatments T1, T4, T6, T7, and Control 2, while the other treatments did not show this type of fermentation. The highest percentage of beans with a slaty appearance was 4% in T1, with treatments T3, T5, T6, and T8 not showing this defect. The presence of mold was observed in almost all treatments, ranging from 1% to 5% of the beans, with treatments T4, T7, and Control 2 not showing any mold, while treatments T4 and T6 showed damage caused by insects.

All samples for both Nacional and CCN-51 cocoa showed values that meet the limits set by the Ecuadorian standard INEN 176:2021 [21] for GF, MF, and S beans. The standard requires a minimum of 75% completely fermented beans, a maximum of 15% medium fermented beans, and a maximum of 9% slaty beans. With regard to beans with mold, it was found that treatments T1, T4, T6, T7, T9, and Control 2 for Nacional cocoa genotype, as well as treatments T4, T7, and Control 2 for CCN-51, complied with the requirements of the standard, which states that the presence of mold should not exceed 1%. Regarding mold damage, the results of the Nacional cocoa were better than those of CCN-51, since the number of Nacional samples with ID was lower, and when values exceeded the limit of 1%, the maximum percentage of beans with mold was 2%. For CCN-51, the majority of the samples showed M, with percentages higher than in Nacional cacao, reaching a maximum of 5% for T3. In this study, when the PTE enzyme was used for fermentation, the incidence of mold in Nacional cocoa was generally lower than that obtained previously [22].

### 2.2. Chemical Analysis

#### 2.2.1. pH and Acidity

**Figure 1:**
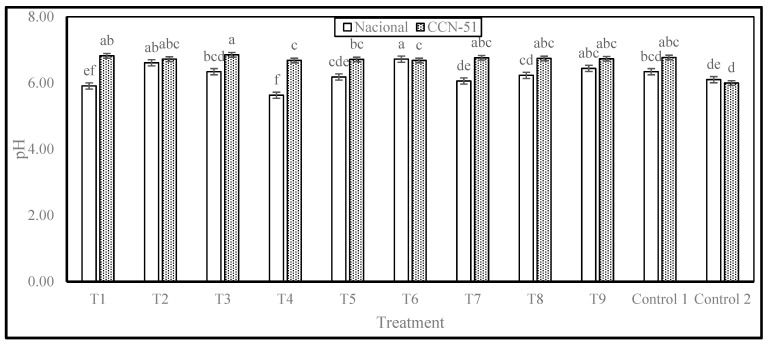
pH results for Nacional and CCN-51 cocoa beans. Note: The results are expressed as the mean ± standard deviation of three replicates. Analyses were performed separately for each genotype, and different letters in columns indicate significant differences (*p* < 0.05) according to Tukey’s test.

The pH values obtained are displayed in Figure 1. Significant differences were identified among the various treatments applied to the Nacional cocoa genotype, with six levels of statistical significance. Treatment T6 exhibited the pH value closest to neutrality (6.72), followed by T2 and T3. Conversely, the T4 treatment exhibited the lowest pH value (5.63), a result that differed significantly from the other samples.

Comparison with the study by Morales et al. (2024) [9] on fine aroma cocoa from the parish of Valle Hermoso reveals that the pH values obtained in that study ranged from 4.72 to 6.08, indicating a higher acidity compared to the results obtained in the present study [9].

The analysis of these results indicates that the pH of Nacional cocoa varies with the treatments applied, influenced by factors such as the dose of PTE, mucilage washing, and drying conditions. A lower pH, as observed in T4, suggests higher acid production during fermentation, which could be associated with higher activity of fermentative microorganisms in the absence of washing and a shorter resting time during pre-drying. Conversely, treatments exhibiting higher pH, such as T6 and T2, might be associated with diminished organic acid accumulation, potentially attributable to a synergetic effect of washing and drying under regulated conditions.

In the CCN-51 cocoa genotype, variations in pH were also observed among the various treatments, with four levels of statistical significance. Treatment T3 exhibited the pH value closest to neutrality (6.85), followed by T1 and Control 1. Conversely, Control 2 exhibited the lowest pH value (6.00), a result that differed significantly from the other samples.

The CCN-51 cocoa beans exhibited higher pH values compared to the Nacional in all treatments. This finding suggests a reduced level of acidification during processing, which may be attributable to variations in chemical composition and microbial activity during fermentation, resulting in diminished production of acidic compounds or augmented buffering capacity within the bean pulp.

These findings have implications for the quality of fermented cocoa, as a higher pH in CCN-51 may contribute to a less acidic and milder sensory profile compared to Nacional cocoa. Furthermore, the disparity between Control 1 and Control 2 underscores the pivotal influence of processing methodology on the ultimate pH of the beans, thereby emphasizing the significance of parameters such as washing, pre-drying, resting time, and drying technique.

**Figure 2:**
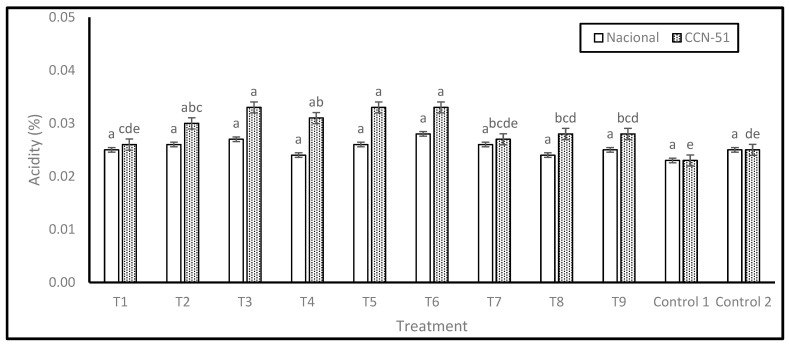
Acidity results for Nacional and CCN-51 cocoa beans. Note: The results are expressed as the mean ± standard deviation of three replicates. Analyses were performed separately for each genotype, and different letters in columns indicate significant differences (*p* < 0.05) according to Tukey’s test.

The acidity results, expressed as a percentage of acetic acid, are shown in Figure 2. For the Nacional genotype, no significant differences were found between the treatments; however, treatment T6 showed the highest acidity percentage (0.03), followed by T3 (0.03), and the lowest acidity value was observed in Control 1 (0.02).

The absence of significant differences suggests that, despite the differences in treatments, the percentage of acidity in the Nacional variety remains relatively stable. This homogeneous response indicates that Nacional variety exhibits a reduced propensity for organic acid accumulation during fermentation, irrespective of factors such as PTE dosage, degree of washing, pre-drying time, or type of drying.

In the case of the CCN-51 genotype, significant differences were identified, with five levels of significance. Treatments T3, T5, and T6 exhibited the highest percentages of acidity (0.03). The treatment regimen encompassed a combination of complete or incomplete washing, pre-drying, resting periods, and drying on various surfaces, as shown in the Experimental Design section. Conversely, treatment T1 (0.03) exhibited the lowest acidity percentage.

#### 2.2.2. Cadmium Concentration

**Figure 3:**
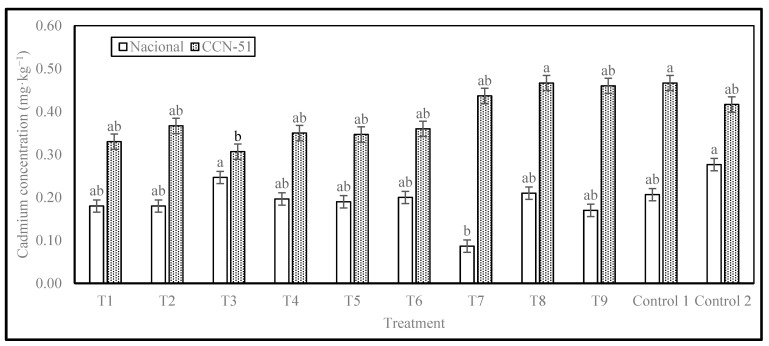
Cadmium concentrations in Nacional and CCN-51 genotypes. Note: The results are expressed as the mean ± standard deviation of three replicates. Analyses were performed separately for each genotype, and different letters in columns indicate significant differences (*p* < 0.05) according to Tukey’s test.

The results of Cd concentration are presented in Figure 3. The Nacional cocoa genotype showed significant differences, with 2 levels of significance. The highest Cd concentration of 0.28 mg·kg^−1^ was observed in Control 2, which consisted of cocoa beans from local producers without enzyme. The lowest concentration was in T7, with an enzyme dose of 0.30 mL·kg^−1^ of cocoa in slime, without washing, with a resting period of 48 h before pre-drying and drying in tents, with a cadmium content of 0.09 mg·kg^−1^, representing a 69% reduction compared to the highest value observed (Control 2).

The CCN-51 genotype also showed significant differences, with 2 levels of significance. T3 treatment had the lowest value, 0.31 mg·kg^−1^, corresponding to the treatment with an enzyme dose of 0.10 mL·kg^−1^ of cocoa in slime, with complete washing, rest in pre-drying for 48 h, and drying on a splint. This result also indicates a reduction of 26.37% in relation to Control 2 (Local producer control, without enzyme). The highest value for CCN-51 was 0.47 mg·kg^−1^ of Cd, obtained for Control 1 (mechanical removal of mucilage before fermentation and drying) and T8 samples (enzyme dose 0.30 mL·kg^−1^ of cocoa in slime, with incomplete washing, without rest in pre-drying, drying on a splint), which represents an increase of 12% in relation to Control 2 (0.42 mg·kg^−1^).

The cadmium concentrations in cocoa beans obtained in this study are lower than those previously obtained by Morales-Rodríguez et al. (2022) in the province of Esmeraldas, which found Cd concentrations ranging from 0.53 to 2.48 mg·kg^−1^ for the Nacional genotype and from 0.40 to 2.72 mg·kg^−1^ for the CCN-51 genotype [17]. Similarly, the results in this research are lower than those reported by Lanza et al. (2016) for cocoa from Venezuela, where Cd concentrations ranging from 0.95 to 2.09 mg·kg^−1^ were recorded [23,24,25]. Furthermore, it was shown that none of the treatments exceeded the permitted limits for chocolate products set by the European Union (0.8 mg·kg^−1^), which was used as a basis by some cocoa bean buyers [15]. This can be attributed to a number of factors, both abiotic and biotic, that have a direct impact on soil, plantation, and fruit quality.

**Figure 4:**
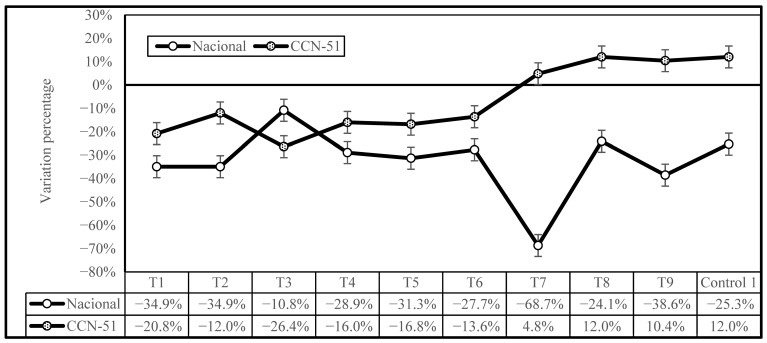
Differences in the percentages of Cd reduction in Nacional and CCN-51 genotypes of cocoa beans subjected to different treatments compared to Control 2.

Figure 4 illustrates the variations in the percentage of Cd reduction due to the different treatments under investigation in comparison to Control 2, showing the effectiveness of the treatments for both genotypes. The most noteworthy outcome is that the treatments were more effective at reducing Cd concentration in the Nacional cocoa genotype than the CCN-51 genotype, with the exception of T3. The T7 treatment was demonstrated to have the highest efficacy for the Nacional genotype samples, followed by T9, T1, and T2, with a mean reduction of 6.6% for the CCN-51 genotype and 32.5% for the Nacional genotype. The effect was different for CCN-51, with samples T7, T8, T9, and Control 1 having an increase in Cd levels, ranging from 4.8% (T7) to 12.0% (T8) compared to Control 2. Therefore, it can be concluded that a higher enzyme concentration is not effective in reducing cadmium levels in CCN-51cocoa beans.

**Figure 5:**
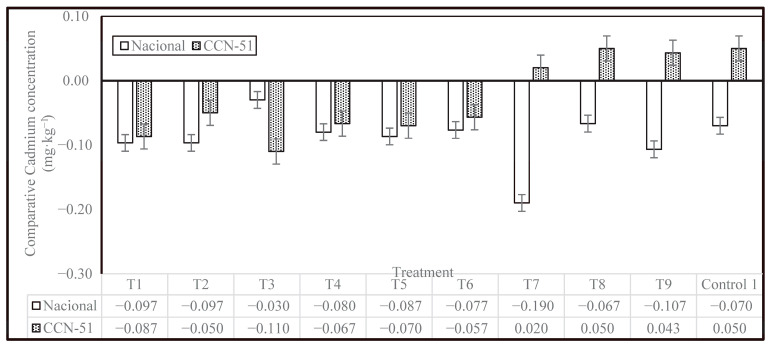
Average reduction in Cd (mg·kg^−1^) in the Nacional and CCN-51 genotypes in relation to Control 2.

Figure 5 shows a comparison of cadmium concentrations in the two cocoa genotypes, Nacional and CCN-51, across different treatments with respect to Control 2. It is observed that, in general, there is a reduction in cadmium levels compared to Control 2, as evidenced by the negative values in most of the treatments; however, the magnitude of the reduction varies depending on the treatment applied and the type of cocoa. The PTE enzyme showed an ability to reduce Cd concentration in cocoa beans, especially at concentrations of 0.1 and 0.2 mL·kg^−1^. The Nacional genotype showed greater reductions in cadmium content than the CCN-51 genotype, with the decrease being more pronounced for the T7 treatment. In contrast, CCN-51 showed a different behavior pattern. In treatments T7, T8, T9, and Control 1, the values were positive, indicating an increase in cadmium concentration with respect to Control 2.

The findings of this study, in conjunction with the results of other research, demonstrate that postharvest practices that are effective in reducing cadmium levels in Nacional may not be equally effective for CCN-51, and there is a possibility that they may even be counterproductive.

### 2.3. Sensory Quality Assessment

The results of the sensory test are presented in Table 2. Significant differences were found in the flavor variables Astringent, Bitter, Cocoa, Floral, and Nutty, as well as the light/dark brown visual appearance of the cocoa beans of the Nacional genotype. For astringency, T8 and Control 2 had high values (8.67 and 7.67, respectively), while the remaining treatments had a value of 0. For bitterness, T5 had the highest value (10), followed by T2 and T6 (8.67) and T3, T4, and T9 (7.67). Control 2 and T8 had the lowest bitterness value (0), as they were the samples with the highest astringency. For Cocoa flavor, T5 (9.33) and T3 (8.67) had the highest values, while four treatments (T2, T7, T8, and Control 1) had a value of 0. Fruity, Floral, and Nutty flavors were absent in almost all treatments except T2 (Floral) and T8, T7, and Control 1 (Nutty). In addition, none of the treatments had an acidic flavor, which is considered undesirable in cocoa. In terms of appearance, the light color predominated in Control 1 and T6, while the dark color predominated in the remaining treatments, with T1 and T9 having the highest value (9.33).

For cocoa beans of the CCN-51 genotype, significant differences were found in the variables analyzed, with the exception of acidity. For Astringent flavor, only Control 2 showed a significantly high value (5.67), while each of the remaining treatments had a value of 0. For the Bitter variable, significantly higher values were obtained for T2 (9.00) and T3 (8.67), while Control 2 had a score of 0. None of the treatments had an acidic flavor, similar to the Nacional beans. In terms of Cocoa flavor, treatments T3 and T4 had the highest value (9.00), while Control 2, T7, and T8 had a value of 0. In terms of Fruity, Floral, and Nutty flavors, Control 2 had a significant value for Fruity flavor (6.17), unlike Nacional beans, where none of the treatments showed fruity flavors. T5 had a high Floral flavor value (8.00), while T7 and T8 treatments had a significant Nutty flavor (6.33 and 5.33). Regarding visual appearance, T4 and T1 had the highest values for light color, while T3 (9.67) had the highest value for dark color. Control 2 had low values for both visual assessment parameters.

The development of aromas and flavors depends both on fermentation and other factors, such as roasting, which enhances the sensory characteristics of the cocoa. The taste of Nacional cocoa is very specific and distinct from other cocoa genotypes; it is described as floral and strong, with hints of astringency or a leguminous character. For cocoa to be classified as first quality, it must develop its characteristic aroma and flavor. These qualities only develop when the beans, properly fermented and dried, are properly roasted [26].

### 2.4. Pearson’s Correlation

The bivariate correlations between the variables analyzed in this research were studied for Nacional cocoa (Table 3). A moderate positive correlation was observed between pH and Medium Fermentation (0.497), indicating that higher pH levels tend to be associated with medium fermentation levels. Conversely, the Mold Presence exhibited a significant negative correlation with pH (−0.346), suggesting that a lower pH could favor mold growth, possibly due to inadequate fermentation conditions or higher humidity.

The findings demonstrated a moderate correlation between Acidity and Over-Fermentation (0.420), indicating that excessive fermentation may result in acid accumulation in the beans. This phenomenon can be attributed to excessive degradation of sugars and other fermentable compounds, resulting in the generation of undesirable secondary acids. A noteworthy observation was the positive correlation between Acidity and the variables Slate (0.375) and Insect Damage (0.498). This finding suggests that cocoa beans with higher acidity levels may be more susceptible to structural defects, potentially resulting from improper fermentation or drying conditions. In terms of color, the more acidic beans tended to be less light brown (−0.345), suggesting a possible relationship between acidity and bean darkening during the fermentation and drying processes. The strong correlation between Acidity and cadmium concentration (0.523) is noteworthy. This finding suggests that more acidic environments may favor the absorption or accumulation of cadmium in beans, which is a significant risk factor in cocoa production, given that cadmium is a heavy metal regulated in many international markets. This phenomenon can be attributed to the influence of Cd on the metabolic processes of microorganisms during fermentation, resulting in increased acid production [27].

The correlation between Good Fermentation and Over Fermentation was strongly positive and significant (0.605), suggesting that the search for optimal fermentation may lead to a risk of over-fermenting grains. There was also a positive correlation between cadmium content and Good Fermentation (0.357), indicating that certain fermentation processes favor the accumulation of this heavy metal in cocoa beans. This is a matter of concern, given that cadmium is a regulated contaminant in the cocoa industry.

An important finding was the strong correlation between Low Fermentation and Insect Damage (0.639), suggesting that grains with insufficient fermentation are more susceptible to insect damage. This may be due to a softer internal structure or less development of antimicrobial compounds during fermentation.

A significant correlation was found between Over Fermentation and Slate grains (0.386), which indicates that excess fermentation may be associated with the appearance of physical defects, such as slate, which affects grain structure. In addition, a strong positive correlation was found with cadmium content (0.574), suggesting that grains may accumulate more cadmium during the overfermentation process. This finding reinforces concerns about the health risks associated with overfermentation, especially with regard to food safety.

Mold Presence had a significant negative correlation with the Dark Brown attribute (−0.493), which suggests that the presence of mold negatively affects cocoa coloration, making it less visually appealing.

A strong correlation was found between Slate beans and Insect Damage (0.581), confirming that beans with structural defects such as slate are more susceptible to insect attack, probably due to their lower physical strength or reduced moisture retention, which may make them more vulnerable.

Bivariate correlations between the variables were obtained for cocoa beans of genotype CCN-51 (Table 4). The pH level was correlated with some fermentation variables and cocoa color, including a positive association with Medium Fermentation (0.444) and a negative association with Good Fermentation (−0.406). This finding suggests that higher pH levels may be indicative of less optimal fermentation conditions. In addition, pH has a strong influence on cocoa color, with a negative correlation with Light Brown color (−0.493) and a positive correlation with Dark Brown color (0.739), which may be due to oxidation reactions.

Acidity exhibited a positive correlation with Medium Fermentation (0.443), indicating its continued perceptibility at intermediate fermentation levels. Furthermore, a positive association was observed between Acidity and Light Brown (0.819) and Dark Brown (0.536) color notes. Additionally, a negative correlation was identified between Acidity and cadmium content (−0.487), suggesting that more acidic beans may contain lower concentrations of this heavy metal.

A strong negative correlation was observed between Good Fermentation and Medium (−0.651) and Low (−0.486) Fermentation, indicating that as optimal fermentation is achieved, the proportion of beans with insufficient fermentation decreases significantly. In addition, a negative correlation was observed between Good Fermentation and Mold Presence (−0.402), suggesting that adequate fermentation helps to reduce the proliferation of fungi in the grains. A particularly notable finding was the strong positive correlation between Good Fermentation and Light Brown color (0.766), while the relationship with Dark Brown color was negative (−0.480). This finding indicates that grains undergoing effective fermentation tend to exhibit a lighter coloration, likely attributable to the degradation of polyphenols and the reduction of compounds responsible for the browning process.

There was also a strong negative correlation between Medium Fermentation and Light Brown color (−0.759), whereas the correlation with Dark Brown color was positive (0.574); this suggests that grains with medium fermentation tend to have a darker shade, possibly due to the retention of phenolic compounds and partial oxidation.

A significant negative correlation between Low Fermentation and Light Brown color (−0.542) suggests that lower fermentation levels lead to a reduced presence of light brown tones in the beans. This aligns with the understanding that well-fermented beans exhibit more pronounced brown hues, while under-fermented beans retain a more purplish or unprocessed appearance.

A strong positive correlation was found between Over Fermentation and Insect Damage (0.601), indicating that over-fermented beans are more likely to be damaged by pests, possibly due to their greater structural deterioration. A negative correlation was observed between Over Fermentation and Dark Brown color (−0.374), indicating that over-fermentation may reduce the intensity of the dark shade characteristic of well-fermented cocoa.

Mold Presence showed a strong negative correlation with Light Brown color (−0.686), indicating that beans with mold tend to lose clarity in hue; in contrast, a positive correlation was found with Dark Brown color (0.403), suggesting that affected beans may have a darker shade, possibly due to degradation of their internal structure. The analysis revealed a significant positive correlation between Insect Damage and Light Brown color (0.542), suggesting that beans affected by insects tended to have a lighter appearance.

Finally, there was a significant positive correlation between Light Brown color and Cadmium level (0.501). This suggests that an increase in cadmium levels in cocoa is associated with a greater presence of light brown tones. This relationship may indicate that cadmium affects the color of cocoa during the drying or fermentation processes, thereby influencing its visual appearance.

### 2.5. Principal Component Analysis

To explain the correlations in the results and provide a better understanding of the question, principal component analysis (PCA) was performed, taking into account the two genotypes, Nacional and CCN-51, and considering the variables pH, Acidity, Good Fermentation, Medium Fermentation, Insect Damage, Bitter and Cocoa flavors, Dark Brown, and Cadmium (Table 5).

The PCA revealed that the first two principal components explained 53.70% of the total variability: Principal Component 1 (PC1) explained 31.10% of the variability, while Principal Component 2 (PC2) explained 22.60% of the variability. PC1 stands out for the expression of the variables Good Fermentation (0.51), which has the highest absolute loading in PC1, followed by Medium Fermentation (−0.49) and Bitter (−0.48), with negative loadings. On the other hand, in PC2, the most important variables were Cadmium (0.58), pH (0.57), and Acidity (0.46), which had the highest absolute loadings.

PC1 has, in its positive component, the variable Good fermentation, and in the negative component, Medium fermentation and Bitter flavor, thus defining a quality axis for the fermentation process. The samples with the highest positive values were Control 1, Control 2, T7, and T8 for the Nacional genotype, and Control 1, Control 2, and T7 for the CCN-51 genotype. On the negative side of the PC1 axis are T3 for CCN-51 and T1 for Nacional with the lowest values.

**Figure 6:**
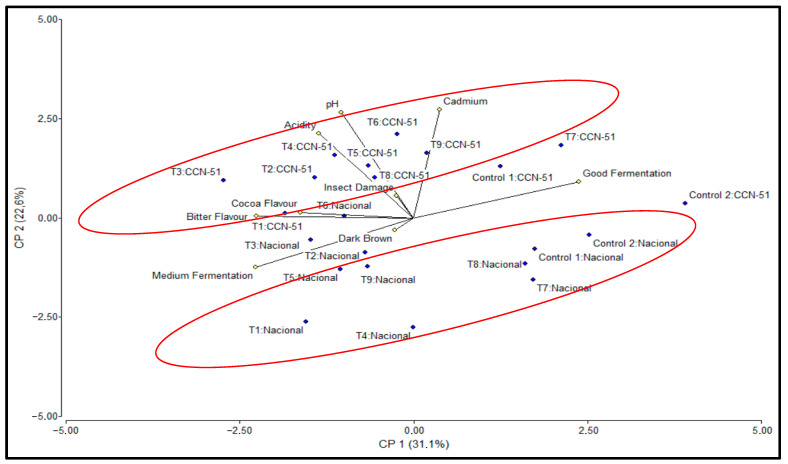
Samples and variables in the Principal Component Analysis plane (PC1: 31.1% of total variance; PC2: 22.6% of total variance) for cocoa beans of Nacional and CCN-51 genotypes.

The CCN-51 samples had positive PC2 values (higher than 0), with higher positions than those of the Nacional genotype in the plane defined by PC1 and PC2, as they have higher Cd contents than the corresponding Nacional cocoa treatments (Figure 6).

## 3. Discussion

The rate of fermentation can be influenced by various external factors, including temperature, process duration, geographical location, and the specific variety of cocoa beans [22]. During the fermentation process, cocoa beans change color because the polyphenolic compounds in the cotyledon are oxidized by the enzyme polyphenol oxidase in the presence of air, changing the color of the bean from purple to brown. If the cotyledon is not fully fermented, it still contains high levels of polyphenols, preventing it from turning brown [28]. Insect damage in the cutting test was very low; the presence of insects in cocoa beans is often a consequence of inadequate postharvest handling that fails to follow Good Handling Practices (GHPs) and Good Manufacturing Practices (GMPs). Insect infestation of cocoa beans causes mechanical damage or injury, as well as the formation of holes, which cause the cocoa beans to break and become susceptible to easy infestation and infection by mold [28].

Regarding pH and acidity, the CCN-51 genotype exhibited higher acidity values than the Nacional genotype in most of the treatments. This finding indicates an increased susceptibility of CCN-51 to acid accumulation during the fermentation process, or alternatively, a heightened extraction of acid precursors resulting from the impact of the applied treatment. An increase in acidity could be associated with higher production of organic acids during fermentation. Rivera et al. (2012) [29] note that the physical and chemical transformations that occur during cocoa fermentation, involving yeasts and enzymes, play a fundamental role in the variation of acidity. Different acids are produced, such as acetic, oleic, and stearic, which not only modify the acidity level but also contribute to the development of characteristic and pleasant flavors in the final product [29].

On the other hand, the Nacional genotype tends to accumulate a lower quantity of cadmium than CCN-51 under analogous fermentation conditions. This phenomenon may be attributable to genetic variations in cadmium uptake, translocation, or storage. Furthermore, certain treatments, such as T7, could be viable options for mitigating cadmium content in the Nacional variety; however, they do not have the same effect in the CCN-51 variety. Thus, the effectiveness of postharvest practices in reducing cadmium levels depends on the cocoa genotype.

Some of the treatments applied reduced cadmium levels, with a similar or higher efficacy than others considered to be more effective [23]. In soils with high levels of Cd, a combination of pre and postharvest strategies may be an effective approach for more effectively reducing Cd concentration in cocoa beans. Durango et al. (2021) evaluated the efficacy of various organic substances in reducing Cd concentrations in cocoa beans across three Ecuadorian provinces [24]. They found that the application of charcoal and cachaza significantly reduced the Cd content in cocoa beans by more than 50% in Santa Elena province. Of the three provinces studied, only the Cd contents of beans from the province of Manabí were similar to those obtained in the present study; in the other provinces, the Cd content of cocoa beans exceeded the tolerable limit established by the EU (0.8 mg·kg^−1^). Another strategy involved the postharvest application of acidifying agents, chelators, and various pre-drying and washing processes on cocoa beans. Guzman (2020) [30] demonstrated that washing and pre-drying processes resulted in a reduction of Cd content by 49.21% and 28.6%, respectively. Among the chelating agents tested, citric acid was identified as the most effective, achieving a 17.1% reduction in Cd levels; furthermore, among the acidic treatments, hydrochloric acid solution with a pH of 3.5 was identified as the most effective during the reception stage, with a reduction in Cd levels of 15.48% [30].

It is important to reduce cadmium levels in cocoa beans, as Cd is transferred into chocolate, as confirmed by Amorello et al. (2025) [31], who evaluated cadmium, lead, and nickel concentrations in 52 different chocolate samples. They concluded that Cd was present in chocolates at concentrations ranging from 37 μg kg^−1^ to 610 μg kg^−1^, with an average concentration of 63 μg kg^−1^. These authors also noted that the estimated daily intake of Cd is 0.015 and 0.071 μg kg^−1^ per day in adults and children, respectively; thus, it is essential to control the cadmium content in raw materials [31].

The generation of sensory data is a highly subjective process; however, the training of judges aims to reduce subjectivity, as evidenced by the low values of standard deviations obtained. The judges’ sense of taste and smell are the instruments directly involved in the sensory measurement, adding a layer of variability and subjectivity to the analysis [32].

The presence of astringent or bitter flavors in cocoa has been associated with high polyphenol content. These components are fundamental to cocoa flavor, being responsible for bitterness and astringency, characteristics that vary based on genotype and environmental conditions. These flavors tend to be more pronounced in cocoa beans with low fermentation, negatively affecting the sensory qualities of the chocolate. Proper fermentation minimizes bitter and astringent flavors, leading to the expression of nutty, fruity, and floral flavors. This allows the chocolate to develop all its characteristic flavors without being masked by the negative attributes associated with insufficient fermentation [33].

Concerning the primary research objective, which was to evaluate the effects of varying doses of PTE enzymes on Cd concentrations, the results demonstrated a substantial reduction in Cd content in response to specific treatments. In Nacional cocoa, treatment T7 (0.30 mL·kg^−1^ PTE) showed the most significant reduction—reaching 68.6%—compared to the control, while in CCN-51 cocoa, treatment T3 (0.10 mL·kg^−1^) exhibited a moderate reduction of 26.4%. These findings are consistent with those reported by Morales et al. (2022), who observed PTE-mediated reductions, albeit with reduced efficiency, in Esmeraldas province. This finding serves to substantiate the fundamental role of enzyme dosage and genotype interaction in the context of cadmium uptake and retention [17].

The second objective of the study was to assess the impact of mucilage washing, resting time, and drying method on Cd levels. Treatments involving extended pre-drying rest and regulated drying (e.g., in a marquee) typically resulted in reduced Cd concentrations. This finding aligns with the conclusions of a previous study by Guzmán (2020), who determined that the pre-drying and washing steps contributed to significant Cd reduction [30]. However, the response varied based on genotype—while Nacional benefited more consistently from these treatments, CCN-51 exhibited inconsistent behavior, and in some cases (T7–T9), Cd levels increased. This underscores the significance of tailoring postharvest strategies to specific genotypes.

Thirdly, regarding the third objective, it was evident that fermentation variables had an influence on the physico-chemical and sensory characteristics. Variations in pH and acidity levels were observed across the various treatments, with a consequential effect on flavor and fermentation levels. The application of treatments involving controlled washing and resting (e.g., T6) resulted in the maintenance of neutral pH and moderate acidity, which was found to be positively correlated with the presence of desirable sensory attributes such as cocoa and nutty flavors. Conversely, elevated acidity and astringency levels were detected in samples that underwent inadequate or excessive fermentation. These findings align with those of Rivera et al. (2012) and Torres et al. (2018), who emphasized that optimal fermentation and acid balance enhance flavor development in cocoa [29,34].

The findings of this study demonstrate that PTE application together with bespoke postharvest practices constitutes an efficacious approach for reducing Cd levels while preserving or enhancing cocoa quality, particularly in the Nacional genotype. However, the genotype-dependent variability observed in CCN-51 suggests a need for further optimization or alternative strategies for this cultivar. Thus, the study provides practical insights for producers and policy-makers aiming to meet international cadmium limits without compromising the market value of Ecuadorian cocoa.

The current study hypothesized that using pectin trans-eliminase (PTE) during the fermentation process, in combination with optimized postharvest practices, could significantly reduce cadmium (Cd) levels in cocoa beans without compromising quality. The results obtained provide substantial support for this hypothesis, particularly in the context of the Nacional genotype from Ecuador.

## 4. Materials and Methods

### 4.1. Plant Material

This study was conducted on the Ecuadorian coast, in the province of Los Ríos, in the Mocache canton, which is located at 1°10′60″ South latitude and 79°30′0″ West longitude. Its land relief is divided into two zones, the savannahs and the hills, with an average altitude of 52 m. The climate is humid tropical with two seasons: the dry season (from June to December) and the rainy season (from December to May). Rainfall varies between 1500 and 2500 mm, and the average temperature is 23 °C [35].

Samples were collected from farms belonging to local producers of Machete, Garabato, and Maculillo. Fine-flavor cocoa pods were harvested from cocoa plants of the Nacional genealogy (varietal name EET-62) with an average plantation age of 6 years in the productive stage. These pods are characterized by their rounded shape and smooth, paired ribs. They are green when immature and yellow when ripe. Each plant produces an average of 19 to 20 pods, and its yield varies from 1150 to 1500 kg per hectare. The beans are round and flattened, large and dark purple in color, with an average weight of 1.6 g per bean [36].

CCN-51 cocoa pods were harvested from cocoa plants of Hybrid triple Forastero genealogy (varietal name (IMC-67 × ICS) × Canelo) with an average plantation age of 6 years in the productive stage. These pods are noted for their oblong shape, paired and textured ribs, and lower incidence of diseases. Their color is purple in the immature stage and orange–red when mature. Each plant produces approximately 16 pods on average, and the yield varies between 1500 and 2760 kg per hectare. The beans are elliptical in shape, large and light purple in color, with an average weight of 1.5 g per bean [36].

### 4.2. Experimental Design

Comparisons between Nacional and CCN-51 genotypes were conducted using a completely randomized design (CRD) with three replications. These trials were combined in an orthogonal arrangement [23]. The Taguchi method, a technique that enables the separation of the effects of factors and levels studied, was applied to analyze the design of a product or process aiming to achieve optimum performance. With the Taguchi method, an experimental sequence that facilitates product improvement is followed [37].

This study is a factorial design with four factors: A (PTE enzyme concentration) × B (types of mucilage washing) × C (pre-drying rest) × D (drying types), each consisting of three levels, equal to 3A (0.10; 0.20; 0.30 mL·kg^−1^ PTE enzyme) × 3B (without washing, incomplete washing and complete mucilage washing) × 3C (without resting, 24 h resting and 48 h resting prior to drying) × 3D (cement line, marquee, splint), to give a total of 81 treatments. If this experiment were to be conducted with three replicates, 243 experimental units would be required, making it technically infeasible. Taguchi’s method was developed to reduce the number of treatments, based on an orthogonal arrangement, while maintaining the validity of the study [25]. In this study, with 81 treatments, the orthogonal design L9 (3)4 is reduced to only 9 treatments, as shown in Table 6.

In addition, two control samples were included for comparison: a control with mechanical removal of mucilage, fermentation, and drying based on the local process (Control 1) and a local producer control (Control 2) obtained directly from a farmer in the canton of Mocache. The control samples had a volume of cocoa beans similar to the other treatments and were not exposed to the PTE enzyme. A total of 11 treatments were set up for each genotype. Table 7 shows the samples and the experimental design.

The field samples consisted of 25 healthy and mature cocoa pods for each genotype. The quantity and weight of the actual dry cocoa sample corresponded to those obtained after postharvest processing.

The cocoa beans were removed from the pods and fermented in wooden boxes measuring 15 cm × 10 cm × 12 cm. Each box held 2 kg of cocoa beans. One box was used per treatment. The PTE enzyme used was a commercial enzyme supplied by Granotec Ecuador S.A. (CAS No. 9033-35-6), lot CCC63-R210132ST (Granotec Ecuador, Guayaquil, Ecuador). The fermentation was conducted for 5 days. The samples were then dried to reduce the moisture content.

### 4.3. Cutting Test

The percentage of fermented beans was assessed using the NTE INEN-ISO 1114:2013 cutting test [38]. The objective of this test is to evaluate the level of fermentation of the cocoa beans and to detect any problems that may have occurred during the process. During this test, 100 randomly selected cocoa beans were cut lengthwise and placed on a wooden board divided into 100 sections to expose the maximum surface area of the cotyledons for visual inspection. Each bean was examined in daylight or under equivalent artificial light. The classification criteria were as follows: Good Fermentation (GF), beans with brown or reddish-brown cotyledons and clear fermentation streaks; Medium Fermentation (MF), beans with slightly striped cotyledons and a faint violet tinge; Low Fermentation (LF), beans with intensely violet cotyledons; Over Fermentation (OF), beans with dark brown cotyledons and undesirable flavor; Slaty (S), unfermented beans with a blackish grey or greenish color and a compact appearance when cut lengthwise; Mold (M), beans showing partial or total deterioration of internal structure due to mold; and Insect Damage (ID), beans with insect damage [22].

### 4.4. Chemical Analysis

Laboratory analyses were conducted at the Soil and Plant Nutrition Laboratory of the Escuela Politécnica de Litoral (ESPOL) in Guayaquil, Ecuador. The determination of Cd concentration was conducted using the inductively coupled plasma optical emission spectroscopy (ICP-OES) method, adapting the techniques described by Flor et al. (2019), Furcal-Beriguete and Torres-Morales (2020), and Segura Lajo and Soto Baldárrago (2015) [39,40,41]. For each treatment, 3 g of cocoa beans were collected in triplicate, ground and homogenized, and then placed in test tubes. Subsequently, 1 mL of a solution of HNO_3_:HClO_4_ (20:1) was added to initiate the digestion process. The samples were then placed in a water bath at 100 °C for one hour to complete the digestion. At the end of this process, the contents of each tube were transferred to 25 mL flasks and diluted with ultrapure water. The samples were filtered into lidded plastic tubes for further analysis by optical emission plasma spectrometry (ICP-OES, ICAP 7200 series, Thermo Fischer Scientific, Waltham, MA, USA). For quantification, two standard solutions of 0.5 ppm and 1 ppm were prepared from a 1000 mg·kg^−1^ standard Cd, and a blank sample was prepared in a 25 mL flask to which a 3% nitric acid and 3% ultrapure water solution were added to construct a calibration curve and obtain the results. The precision and accuracy of cadmium determination by ICP-OES were validated through replicate analysis and spike–recovery tests. The method demonstrated a limit of detection (LOD) of 0.005 mg·kg^−1^ and a limit of quantification (LOQ) of 0.015 mg·kg^−1^, consistent with values reported in the literature for food matrix analysis. The recovery rates exhibited a range between 93% and 105%, while the relative standard deviation (RSD) remained below 5% for all replicates. The findings of this study corroborate the validity of the analytical methodology employed and are consistent with the results reported by Barreca et al. (2023) in their investigation into trace element intake from traditional Mediterranean meals [42].

The pH level was measured using the AOAC 970.21 method and the OHAUS-Starter 3100 pH meter (Parsippany, NJ, USA). For this, 5 g of cocoa powder was weighed into a 250 mL beaker; then, 45 mL of boiling distilled water was added, the mixture cooled and filtered through a 9 µm filter paper, and the pH of the filtrate (*n* = 3) measured [43]. To ensure the reliability of the titratable acidity measurements, the precision and accuracy of the method were evaluated. All samples were analyzed in triplicate, with a relative standard deviation (RSD) of less than 3%, indicating good repeatability. Accuracy was confirmed using standard solutions and procedural blanks, with recovery rates ranging from 95% to 102%. The procedure followed the guidelines of the AOAC Official Method [42], which is widely accepted for determining the titratable acidity of plant-derived food products. These results confirm that the reported acidity values are precise and accurate within the method’s working range.

The titratable acidity was determined according to the method described by Morales-Rodríguez et al. (2024) [22]. Briefly, 10 g of ground cocoa beans was mixed with 90 mL of distilled water. This suspension was titrated using a 0.1 N NaOH solution of analytical grade. During the titration, 1 mL of 1% phenolphthalein indicator was added to the sample while stirring. The results were expressed as a percentage of acetic acid as the predominant acid.

### 4.5. Sensory Quality Assessment

For each test, 500 g of fine-flavor cocoa beans were weighed. To avoid defects and obtain homogeneous roasting, cocoa beans of uniform size were selected, thus eliminating impurities. Roasting was conducted in clay pots at 115 °C for 20 min. The beans were then ground into a fine paste, known as liquor, which was then subjected to a conching process for 4 h to remove certain compounds and refine the product. This refined and fluid cocoa liquor was used for sensory analysis. It was poured into plastic containers, with each sample given an identification code. Finally, it was cooled and stored under refrigeration until tasting.

The analysis was conducted in triplicate, following the Colombian Technical Guide GTC-165-Sensory Analysis, the Colombian Technical Standard NTC 3929, and using the terminology in the Voltz Glossary [44,45,46]. The cocoa liquor samples were heated in a water bath at a temperature between 40 and 45 °C. The panelists, trained at the State Technical University of Quevedo (UTEQ) and the National Autonomous Institute of Agricultural Research (INIAP), comprised 8 selected experts. Each was provided with 11 samples of each studied genotype (*n* = 3), resulting in a total of 33 samples per genotype for each panelist. They sampled a small amount of liqueur with a wooden paddle, spread it evenly on the tongue, and held it in the mouth for 15 to 20 s, allowing them to identify the olfactory and gustatory characteristics.

The tasting was conducted individually, with breaks to allow the panelists to neutralize the flavors of the previous samples and to minimize sensory fatigue. The evaluations were conducted in separate sessions, one for each genotype. For flavor ratings, the scale and category method was used, specifically the C scale [44,45]. This approach can produce results with an ordinal measurement scale, which means that the numerical values do not have equal intervals. On this scale, higher values represent higher perceived intensities or levels of pleasure; however, a numerical value of zero does not necessarily indicate a total absence of intensity. This is because in ordinal sensory scales, zero can serve as a relative reference point on the scale, as these are subjective perceptions.

Two types of flavor profiles were used in the cocoa liquor tasting evaluation: basic and specific, as described by Torres et al. (2018) [34]. These profiles were scored on a scale from 0 to 10, ranging from complete absence (score 0), through low (0.01–2.99), medium (3.00–5.99), high (6.00–8.99), and very high (9–10) intensities for each flavor (Table 8).

In addition, a visual evaluation of the cocoa liquor was conducted in conjunction with the sensory evaluation, focusing on determining its appearance in terms of light and dark brown shades, as outlined in the following methodology. First, an equal quantity of cocoa liquor is poured into white dishes to ensure accurate evaluation. It is essential that this evaluation is conducted under natural light conditions or standard 5500 K lighting to avoid any distortion in color perception. The evaluation process begins with the distribution of the samples, which are placed on white plates with codes to prevent any bias. Each panelist examines the samples individually, using a color guide containing light and dark brown tones as a reference. The panelists compare each sample to the color guide and record their perception. As for taste, a rating scale of 0 to 10 is used, ranging from no intense color (value 0), through low (0.01–2.99), medium (3.00–5.99), high (6.00–8.99), and very high (9–10) intensities of each color (light and dark brown).

### 4.6. Statistical Analysis

Statistical analysis was conducted using various techniques. Analysis of variance (ANOVA) was used to compare the variances between the means of the different groups, thus allowing the influence of each factor on the response to be determined. In addition, Tukey’s test at 5% was used to perform the separation of means [9,47]. Descriptive statistical analysis was also conducted by creating bar and line graphs. These graphs are used to summarize and analyze datasets, making it easier to understand their basic characteristics. In addition, Pearson’s correlation was used to assess the linear relationship between two quantitative variables. This measure provides information on the strength and direction of the association between variables [22].

Finally, multivariate analysis was conducted using Principal Component Analysis (PCA). This technique enables the reduction of data dimensionality by transforming a set of correlated variables into a smaller set of uncorrelated variables, known as principal components. Interpretation of the results was facilitated by the use of biplot graphs [22].

IBM SPSS Statistics 20 (SPSS Inc., Chicago, IL, USA) and Infostat version 202l (Universidad Nacional de Córdoba, Córdoba, Argentina) statistical software were used to perform these analyses.

## 5. Conclusions

The present study demonstrates that the application of PTE enzyme during the fermentation of cocoa beans, in addition to other factors such as mucilage washing methods, pre-drying resting periods, and drying techniques, can significantly reduce cadmium concentrations. The efficiency of cadmium reduction is genotype-dependent. Higher cadmium reduction was observed for the Nacional cocoa genotype compared to the CCN-51 genotype. The most efficacious treatment was T7, with a dose of 0.30 mg·kg^−1^ of PTE enzyme for the Nacional genotype. For the CCN-51 genotype, the T3 treatment, comprising a dose of 0.10 mg·kg^−1^ of enzyme, yielded the most favorable outcome. The most effective treatment, irrespective of genotype, was T1, with a PTE dose of 0.10 mL·kg^−1^. Compared to the findings of previously reported studies, the results obtained in this investigation are more favorable, indicating a greater efficacy in reducing cadmium.

Utilization of the PTE enzyme in cocoa fermentation in conjunction with the other controlled variables may facilitate the development of more sustainable agricultural practices by reducing the content of cadmium in the final product. This could be particularly advantageous in regions where soil contamination is a significant issue, and will facilitate global trade by ensuring that cocoa products meet the strict cadmium limits set by the European Union and other international markets. The study demonstrates that the implementation of PTE enzyme treatment in cocoa processing not only enhances product quality but also safeguards human health by reducing exposure to cadmium, a toxic heavy metal.

## Figures and Tables

**Table 1:** Cutting test results for Nacional and CCN-51 cocoa beans.

Cocoa Treatment	Degree of Fermentation and Defects (%)						
	GF	MF	LF	OF	S	M	ID
Cocoa Nacional							
T1	78.78 ± 1.92 ab	15.56 ± 1.93 a	2.22 ± 1.92 b	0.00 ± 0.00 a	3.33 ± 0.00 a	0.00 ± 0.00 a	0.00 ± 0.00 a
T2	82.22 ± 1.92 ab	13.33 ± 0.00 ab	2.22 ± 1.92 b	0.00 ± 0.00 a	0.00 ± 0.00 a	2.22 ± 1.92 a	0.00 ± 0.00 a
T3	77.78 ± 1.92 b	10.00 ± 0.00 abc	4.44 ± 1.93 ab	1.11 ± 1.92 a	3.33 ± 3.34 a	2.22 ± 1.92 a	1.11 ± 1.92 a
T4	80.00 ± 3.33 ab	10.00 ± 3.33 abc	10.00 ± 3.33 a	0.00 ± 0.00 a	0.00 ± 0.00 a	0.00 ± 0.00 a	0.00 ± 0.00 a
T5	82.22 ± 5.09 ab	10.00 ± 3.33 abc	2.22 ± 1.92 b	2.22 ± 1.92 a	2.22 ± 1.92 a	1.11 ± 1.92 a	0.00 ± 0.00 a
T6	84.44 ± 1.93 ab	11.11 ± 1.92 abc	2.22 ± 1.92 b	0.00 ± 0.00 a	2.22 ± 1.92 a	0.00 ± 0.00 a	0.00 ± 0.00 a
T7	85.56 ± 1.93 ab	5.56 ± 1.93 c	5.56 ± 1.93 ab	1.11 ± 1.92 a	2.22 ± 1.92 a	0.00 ± 0.00 a	0.00 ± 0.00 a
T8	83.33 ± 0.00 ab	10.00 ± 3.33 abc	2.22 ± 1.92 b	1.11 ± 1.92 a	2.22 ± 1.92 a	1.11 ± 1.92 a	1.11 ± 1.92 a
T9	83.33 ± 3.34 ab	11.11 ± 1.92 abc	3.33 ± 0.00 b	1.11 ± 1.92 a	1.11 ± 1.92 a	0.00 ± 0.00 a	0.00 ± 0.00 a
Control 1	87.78 ± 6.94 ab	6.67 ± 3.34 bc	1.11 ± 1.92 b	1.11 ± 1.92 a	1.11 ± 1.92 a	2.22 ± 1.92 a	0.00 ± 0.00 a
Control 2	88.89 ± 5.09 a	5.55 ± 3.85 c	0.00 ± 0.00 b	3.33 ± 0.00 a	2.22 ± 1.92 a	0.00 ± 0.00 a	0.00 ± 0.00 a
Cocoa CCN-51							
T1	76.67 ± 0.00 a	12.22 ± 1.92 a	3.33 ± 0.00 a	1.11 ± 1.92 a	4.44 ± 1.93 a	2.22 ± 1.92 a	0.00 ± 0.00 a
T2	80.00 ± 6.67 a	8.89 ± 1.92 ab	5.56 ± 5.09 a	0.00 ± 0.00 a	1.11 ± 1.92 a	4.44 ± 1.93 a	0.00 ± 0.00 a
T3	77.78 ± 1.92 a	12.22 ± 1.92 a	4.44 ± 1.93 a	0.00 ± 0.00 a	0.00 ± 0.00 a	5.55 ± 3.85 a	0.00 ± 0.00 a
T4	84.44 ± 1.93 a	10.00 ± 3.33 ab	2.22 ± 1.92 a	1.11 ± 1.92 a	1.11 ± 1.92 a	0.00 ± 0.00 a	1.11 ± 1.92 a
T5	83.33 ± 3.34 a	10.00 ± 0.00 ab	5.56 ± 5.09 a	0.00 ± 0.00 a	0.00 ± 0.00 a	1.11 ± 1.92 a	0.00 ± 0.00 a
T6	85.56 ± 8.39 a	6.67 ± 0.00 ab	3.33 ± 3.34 a	1.11 ± 1.92 a	0.00 ± 0.00 a	2.22 ± 1.92 a	1.11 ± 1.92 a
T7	90.00 ± 3.33 a	4.44 ± 1.93 ab	3.33 ± 3.34 a	1.11 ± 1.92 a	1.11 ± 1.92 a	0.00 ± 0.00 a	0.00 ± 0.00 a
T8	80.00 ± 3.33 a	11.11 ± 5.09 ab	4.44 ± 1.93 a	0.00 ± 0.00 a	0.00 ± 0.00 a	4.44 ± 1.93 a	0.00 ± 0.00 a
T9	84.44 ± 1.93 a	6.66 ± 5.77 ab	2.22 ± 3.85 a	0.00 ± 0.00 a	2.22 ± 1.92 a	4.44 ± 5.09 a	0.00 ± 0.00 a
Control 1	87.78 ± 1.92 a	5.56 ± 1.93 ab	2.22 ± 1.92 a	0.00 ± 0.00 a	2.22 ± 1.92 a	2.22 ± 1.92 a	0.00 ± 0.00 a
Control 2	90.00 ± 8.82 a	3.33 ± 3.34 b	3.33 ± 3.34 a	1.11 ± 1.92 a	2.22 ± 3.85 a	0.00 ± 0.00 a	0.00 ± 0.00 a

The results are expressed as the mean ± standard deviation of three replications. Analyses were performed separately for each genotype. Different letters in the same column indicate significant differences (*p* < 0.05) according to Tukey’s test.

**Table 2:** Results of sensory analysis and appearance of Nacional and CCN-51 cocoa beans.

Cocoa Treatment	Basic and Specific Flavors							Visual Evaluation	
	Astringent	Bitter	Acid	Cocoa	Fruity	Floral	Nutty	Light Brown	Dark Brown
Nacional									
T1	0.00 ± 0.00 c	7.33 ± 0.58 ab	0.00 ± 0.00	8.00 ± 1.00 ab	0.00 ± 0.00	0.00 ± 0.00 b	0.00 ± 0.00 c	0.00 ± 0.00 c	9.33 ± 0.58 a
T2	0.00 ± 0.00 c	8.67 ± 1.15 a	0.00 ± 0.00	0.00 ± 0.00 d	0.00 ± 0.00	4.33 ± 1.15 a	0.00 ± 0.00 c	9.33 ± 0.58 a	0.00 ± 0.00 b
T3	0.00 ± 0.00 c	7.67 ± 0.58 ab	0.00 ± 0.00	8.67 ± 0.58 ab	0.00 ± 0.00	0.00 ± 0.00 b	0.00 ± 0.00 c	0.00 ± 0.00 c	8.67 ± 0.58 a
T4	0.00 ± 0.00 c	7.67 ± 2.08 ab	0.00 ± 0.00	5.67 ± 1.15 c	0.00 ± 0.00	0.00 ± 0.00 b	0.00 ± 0.00 c	0.00 ± 0.00 c	8.33 ± 1.53 a
T5	0.00 ± 0.00 c	10.00 ± 0.00 a	0.00 ± 0.00	9.33 ± 0.58 a	0.00 ± 0.00	0.00 ± 0.00 b	0.00 ± 0.00 c	0.00 ± 0.00 c	7.33 ± 2.52 a
T6	0.00 ± 0.00 c	8.67 ± 0.58 a	0.00 ± 0.00	8.00 ± 0.00 ab	0.00 ± 0.00	0.00 ± 0.00 b	0.00 ± 0.00 c	8.00 ± 0.00 b	0.00 ± 0.00 b
T7	0.00 ± 0.00 c	5.00 ± 3.00 b	0.00 ± 0.00	0.00 ± 0.00 d	0.00 ± 0.00	0.00 ± 0.00 b	6.00 ± 1.73 b	0.00 ± 0.00 c	7.33 ± 2.52 a
T8	8.67 ± 0.58 a	0.00 ± 0.00 c	0.00 ± 0.00	0.00 ± 0.00 d	0.00 ± 0.00	0.00 ± 0.00 b	6.67 ± 0.58 b	0.00 ± 0.00 c	7.33 ± 0.58 a
T9	0.00 ± 0.00 c	7.67 ± 1.15 ab	0.00 ± 0.00	7.67 ± 1.15 ab	0.00 ± 0.00	0.00 ± 0.00 b	0.00 ± 0.00 c	0.00 ± 0.00 c	9.33 ± 1.15 a
Control 1	0.00 ± 0.00 c	7.33 ± 0.58 ab	0.00 ± 0.00	0.00 ± 0.00 d	0.00 ± 0.00	0.00 ± 0.00 b	8.67 ± 0.58 a	9.00 ± 0.00 a	0.00 ± 0.00 b
Control 2	7.67 ± 0.58 b	0.00 ± 0.00 c	0.00 ± 0.00	7.00 ± 0.00 bc	0.00 ± 0.00	0.00 ± 0.00 b	0.00 ± 0.00 c	0.00 ± 0.00 c	8.33 ± 0.58 a
CCN-51									
T1	0.00 ± 0.00 b	7.67 ± 0.58 ab	0.00 ± 0.00	8.67 ± 0.58 ab	0.00 ± 0.00 b	0.00 ± 0.00 b	0.00 ± 0.00 b	8.67 ± 0.58 b	0.00 ± 0.00 d
T2	0.00 ± 0.00 b	9.00 ± 1.00 a	0.00 ± 0.00	7.33 ± 0.58 c	0.00 ± 0.00 b	0.00 ± 0.00 b	0.00 ± 0.00 b	0.00 ± 0.00 c	8.33 ± 0.58 abc
T3	0.00 ± 0.00 b	8.67 ± 0.58 a	0.00 ± 0.00	9.00 ± 0.00 a	0.00 ± 0.00 b	0.00 ± 0.00 b	0.00 ± 0.00 b	0.00 ± 0.00 c	9.67 ± 0.58 a
T4	0.00 ± 0.00 b	8.33 ± 0.58 ab	0.00 ± 0.00	9.00 ± 0.00 a	0.00 ± 0.00 b	0.00 ± 0.00 b	0.00 ± 0.00 b	9.67 ± 0.58 a	0.00 ± 0.00 d
T5	0.00 ± 0.00 b	8.00 ± 1.00 ab	0.00 ± 0.00	0.00 ± 0.00 d	0.00 ± 0.00 b	8.00 ± 1.00 a	0.00 ± 0.00 b	0.00 ± 0.00 c	9.00 ± 0.00 ab
T6	0.00 ± 0.00 b	6.00 ± 1.00 ab	0.00 ± 0.00	7.50 ± 0.50 bc	0.00 ± 0.00 b	0.00 ± 0.00 b	0.00 ± 0.00 b	0.00 ± 0.00 c	6.67 ± 1.53 bc
T7	0.00 ± 0.00 b	5.33 ± 1.53 ab	0.00 ± 0.00	0.00 ± 0.00 d	0.00 ± 0.00 b	0.00 ± 0.00 b	6.33 ± 1.53 a	0.00 ± 0.00 c	7.00 ± 1.00 abc
T8	0.00 ± 0.00 b	7.00 ± 1.00 ab	0.00 ± 0.00	0.00 ± 0.00 d	0.00 ± 0.00 b	0.00 ± 0.00 b	5.33 ± 0.58 a	0.00 ± 0.00 c	9.00 ± 0.00 ab
T9	0.00 ± 0.00 b	6.00 ± 0.00 ab	0.00 ± 0.00	8.00 ± 1.00 abc	0.00 ± 0.00 b	0.00 ± 0.00 b	0.00 ± 0.00 b	0.00 ± 0.00 c	7.50 ± 0.50 abc
Control 1	0.00 ± 0.00 b	6.00 ± 2.65 ab	0.00 ± 0.00	8.00 ± 0.00 abc	0.00 ± 0.00 b	0.00 ± 0.00 b	0.00 ± 0.00 b	0.00 ± 0.00 c	6.00 ± 2.00 c
Control 2	5.67 ± 1.15 a	0.00 ± 0.00 c	0.00 ± 0.00	0.00 ± 0.00 d	6.17 ± 1.26 a	0.00 ± 0.00 b	0.00 ± 0.00 b	0.00 ± 0.00 c	2.67 ± 1.53 d

The results are expressed as the mean ± standard deviation of three replications. Analyses were performed separately for each genotype. Different letters in the same column indicate significant differences (*p* < 0.05) according to Tukey’s test.

**Table 3:** Pearson product-moment correlations of physico-chemical and sensory variables of cocoa beans (Nacional genotype).

Variables	pH	AC	GF	MF	LF	OF	MP	S	ID	LB	DB	Cd
pH	1											
Acidity (AC)	0.245	1										
Good Fermentation (GF)	0.250	0.050	1									
Medium Fermentation (MF)	0.497 **	0.207	−0.275	1								
Low Fermentation (LF)	0.198	0.283	−0.251	0.311	1							
Over Fermentation (OF)	0.150	0.420 *	0.605 **	−0.011	−0.021	1						
Mold Presence (MP)	−0.346 *	−0.261	0.111	−0.027	−0.218	0.077	1					
Slate (S)	0.269	0.375 *	−0.098	0.195	0.176	0.386 *	−0.236	1				
Insect damage (ID)	0.187	0.498 **	−0.070	0.018	0.639 **	0.260	−0.231	0.581 **	1			
Light Brown (LB)	−0.200	−0.345 *	−0.030	0.047	−0.201	−0.001	0.258	−0.169	−0.245	1		
Dark Brown (DB)	0.089	0.203	0.016	0.152	−0.173	0.123	−0.493 **	0.232	0.096	0.332	1	
Cadmium (Cd)	0.090	0.523 **	0.357 *	−0.023	0.014	0.574 **	−0.093	0.334	0.206	−0.296	−0.049	1

Critical values: r_0.05; 31 GL_ = 0.3442 and r_0.01; 31 GL_ = 0.4428. NOTE: * Correlation at the 95% confidence level; ** Correlation at the 99% confidence level.

**Table 4:** Pearson product-moment correlations of physico-chemical and sensory variables of cocoa beans (CCN-51 genotype).

Variables	pH	AC	GF	MF	LF	OF	MP	S	ID	LB	DB	Cd
pH	1											
Acidity (AC)	0.261	1										
Good Fermentation (GF)	−0.406 *	−0.262	1									
Medium Fermentation (MF)	0.444 **	0.443 **	−0.651 **	1								
Low Fermentation (LF)	0.076	0.035	−0.486 **	0.016	1							
Over Fermentation (OF)	−0.152	−0.074	−0.119	−0.177	−0.044	1						
Mold Presence (MP)	0.307	0.159	−0.402 *	0.011	0.035	−0.167	1					
Slate (S)	−0.129	−0.394 *	−0.265	0.074	−0.128	0.145	−0.150	1				
Insect damage (ID)	0.030	0.193	−0.159	−0.106	0.120	0.601 **	−0.070	−0.167	1			
Light Brown (LB)	−0.493 **	0.819 **	0.766 **	−0.759 **	−0.542 **	0.343	−0.686 **	−0.294	0.542 **	1		
Dark Brown (DB)	0.739 **	0.536 **	−0.480 **	0.574 **	0.131	−0.374 *	0.403 *	−0.223	−0.207	0.091	1	
Cadmium (Cd)	−0.082	−0.487 **	0.317	0.376	0.003	−0.044	−0.166	0.092	−0.094	0.501 **	−0.292	1

Critical values: r_0.05; 31 GL_ = 0.3442 and r_0.01; 31 GL_ = 0.4428. NOTE: * Correlation at the 95% confidence level; ** Correlation at the 99% confidence level.

**Table 5:** Eigenvectors of Principal Components.

Variables	PC1	PC2
pH	−0.22	0.57
Acidity	−0.29	0.46
Good Fermentation	0.51	0.19
Medium Fermentation	−0.49	−0.27
Insect Damage	−0.05	0.12
Bitter Flavor	−0.48	0.01
Cocoa Flavor	−0.35	0.03
Dark Brown	−0.06	−0.07
Cadmium	0.08	0.58

**Table 6:** Orthogonal array matrix of the experimental design based on the Taguchi method.

Treatments	Factor A (PTE Enzyme Concentration (mL·kg^−1^)	Factor B (Types of Mucilage Washing)	Factor C (Pre-Drying Rest)	Factor D (Drying Types)
1	0.10	Without washing	Without resting	Cement line
2	0.10	Incomplete washing	24 h	Marquee
3	0.10	Complete washing	48 h	Splint
4	0.20	Without washing	24 h	Splint
5	0.20	Incomplete washing	48 h	Cement line
6	0.20	Complete washing	Without resting	Marquee
7	0.30	Without washing	48 h	Marquee
8	0.30	Incomplete washing	Without resting	Splint
9	0.30	Complete washing	24 h	Cement line

**Table 7:** Trials conducted using Nacional and CCN-51 cocoa genotypes.

Trials	Code	Description
T1	A1B1C1D1	PTE 0.10 mL·kg^−1^ of cocoa in slime, without washing, without rest in pre-drying, and drying on a cement line.
T2	A1B2C2D2	PTE 0.10 m L·kg^−1^ of cocoa in slime, with incomplete washing, rest in pre-drying for 24 h, and drying in a marquee.
T3	A1B3C3D3	PTE 0.10 m L·kg^−1^ of cocoa in slime, with complete washing, rest in pre-drying for 48 h, and drying on a splint
T4	A2B1C2D3	PTE 0.20 mL·kg^−1^ of cocoa in slime, without washing, rest in pre-drying for 24 h, and drying on a splint.
T5	A2B2C3D1	PTE 0.20 mL·kg^−1^ of cocoa in slime, with incomplete washing, rest in pre-drying for 48 h, and drying on a cement line.
T6	A2B3C1D2	PTE 0.20 mL·kg^−1^ of cocoa in slime, with complete washing, without rest in pre-drying, and drying in a marquee.
T7	A3B1C3D2	PTE 0.30 mL·kg^−1^ of cocoa in slime, without washing, rest in pre-drying for 48 h, and drying in a marquee.
T8	A3B2C1D3	PTE 0.30 mL·kg^−1^ of cocoa in slime, with incomplete washing, without rest in pre-drying, and drying on a splint.
T9	A3B3C2D1	PTE 0.30 mL·kg^−1^ of cocoa in slime, with complete washing, rest in pre-drying for 24 h, and drying on a cement line.
Control 1	Mechanical mucilagination	Mechanical removal of mucilage, fermentation, and drying, based on local processes.
Control 2	Local producer control	Producer local control, commercial cocoa obtained from farmers in the area.

**Table 8:** Basic and specific flavors attributes of cocoa liquor, according to Torres et al. (2018) [34].

Attribute: Basic (B) and Specific (S) Flavors	Description
Astringent (B)	You experience an intense sensation of dryness in the mouth, which is felt in all areas, including the tongue, throat, and even the teeth.
Bitter (B)	The sensation is felt on the back of the tongue and throat, and the taste is intense and distinctive, similar to coffee.
Acid (B)	The sensation perceived on the sides and in the center of the tongue can be associated with the flavors of citrus fruits and vinegar.
Cocoa (S)	The characteristic taste resembles that of chocolate, specifically cocoa beans that have been well fermented, dried, roasted, and free of defects.
Fruity (S)	The flavor evokes ripe fruits and is described as a sweet aroma that delights the taste senses.
Floral (S)	You experience a pleasant taste that resembles the aroma of flowers.
Nutty (S)	Resembles the taste of an almond or nut.

## Data Availability

The data presented in this study are available within the article and on request from the corresponding author.
